# Optimizing performance and mood state in competitive swimmers through tapering strategies

**DOI:** 10.3389/fpsyg.2024.1307675

**Published:** 2024-01-23

**Authors:** Hajer Aouani, Sofiene Amara, Haithem Rebai, Tiago M. Barbosa, Roland van den Tillaar

**Affiliations:** ^1^Research Laboratory Sports Performance Optimization (LR09SEP01), National Center of Medicine and Science in Sports (CNMSS), Tunis, Tunisia; ^2^Higher Institute of Sport and Physical Education of Ksar-Said, University of La Manouba, Tunis, Tunisia; ^3^Research Unit (UR17JS01) Sports Performance, Health & Society, Higher Institute of Sport and Physical Education of Ksar Saîd, Universite de la Manouba, Tunis, Tunisia; ^4^Research Centre for Active Living and Wellbeing (LiveWell), Instituto Politécnico de Bragança, Bragança, Portugal; ^5^Department of Sports Sciences, Instituto Politécnico de Bragança, Bragança, Portugal; ^6^Department of Sport Sciences and Physical Education, Nord University, Levanger, Norway

**Keywords:** fatigue, vigor, total mood disturbance, volume training, mental state

## Abstract

Tapering is a concept that is of great importance in relation to performance, due of its great effect on the psychological and physical condition of the swimmer. Therefore, the present study aims to investigate the effect of two-week of tapering characterized by a progressive training volume reduction on mood state and swimming performance in competitive swimmers. Twenty-four competitive male swimmers were randomly assigned into two groups. Experimental group (age = 16.9 ± 0.5 years) and control group (16.1 ± 0.4 years). The mood subscales (tension, depression, anger, fatigue, confusion and vigor), total mood disturbance and swimming performance (50-m front crawl) were measured in pre and posttest. Our findings revealed a significant improvement in mood subscales (20.8 to 47.8%), total mood disturbance (14.4%) and in swimming performance (3.5%) after 2 weeks of tapering training. A significant correlation was observed between the total mood disturbance and the 50 m front crawl (*r* = −0.63) only in the experimental group. It was concluded that a progressive reduction in training volume with a maintain of intensity could improve mood state and swimming performance. In addition, a change in total mood disturbance could affect swimming performance. Swimming coaches are advised to include tapering period according to the standards we mentioned earlier before competitive swimming to improve mental state, which helps the swimmers to overcome the negative influences of overtraining and therefore they can promote sprint-swimming performance.

## Introduction

For optimal performance on the day of a competitive swim meet, swimmers go through periods of preparatory training (i.e., aquatic swimming training, dry-land training) with the goal of improving several physical abilities (e.g., muscle strength, aerobic capacity and anaerobic capacity) (Amara et al., 2023; Monteiro et al., 2023). Therefore, some negative effects (e.g., muscle fatigue, mental fatigue and injury) may appear during these preparatory training sessions (De Martino and Rodeo, 2018; Chortane et al., 2022). For this reason, tapering is a common training strategy used to reduce fatigue and improve athletic performance (Bachelor et al., 2023; Stone et al., 2023). In fact, up until now, tapering strategies in swimming require even more insight and information (e.g., optimal training volume and intensity, training frequency) and more specifically their effects on psychological state of competitive swimmers. For this reason, current research highlights the study of the effect of a two-week of tapering on psychological state in competitive swimming.

Tapering period (1 to 3 weeks) positively affected psychological status (i.e., mood, wellness, anxiety, and stress) in several sports (e.g., rugby, cycling, triathletes, and tennis) (O’Connor et al., 1989; Coutts et al., 2007a,b). Reduction of psychological and physical fatigue after tapering may be an important factor in improving the mental state of competitive athletes (Bachelor et al., 2023; Stone et al., 2023). In addition, a tapering period (2-week) characterized by a progressive decrease in training volume and maintenance of training intensity resulted in a decrease in rating of perceived exertion (RPE, 25.3%), which, improved perceived ratings of wellness (25.6 to 32.5%) and 20-m swimming performance (2.1%) [10] (Botonis et al., 2019). In the same context, a meta-analysis developed by Bosquet et al. (2007) indicated that 8 to14 day progressive reduction in training volume of 41 to 60%, which maintains training intensity and frequency, was the optimal strategy of tapering for most swimmers and runners. In another context, standard training in swimming (e.g., aerobic and anaerobic training, high-volume training), and as in other sports, can cause negative effects on the psychological state, which requires the implementation of a tapering period in training strategies to reduce the negative effects of standard training (Chortane et al., 2022).

In swimming, a tapering period (7 to 21-days) is found beneficial for improving swimming performance (Houmard et al., 1994; Papoti et al., 2007; Botonis et al., 2019). More specifically, tapering could improve muscle adaptation (i.e., gain in force transfer, muscle power), which helps the swimmer to optimize his swimming technique and be faster in the water (Mujika et al., 1995; Amara et al., 2021). In general, most of the previous scientific studies has discussed the trajectories of swimming performance after the tapering period at physiological and physical levels, while it is also very important to see how the psychological state affects the swimming performance after the tapering period (Mujika et al., 1995; Amara et al., 2021, 2023).

Mood state is a very frequent factor in the assessment of psychological state in competitive athletes (Bijukumar, 2021; Selmi et al., 2023; Stone et al., 2023). Several factors can affect mood such as training load (i.e., volume, intensity and frequency of training) (Selmi et al., 2020; Fields et al., 2021) In fact, physiological (i.e., muscular and cardiorespiratory adaptation, energy) and mental (i.e., concentration, attention and alertness) responses could affect mood state in competitive athletes (Beedie et al., 2000; Hashim and Hanafi Ahmad Yusof, 2011; Bijukumar, 2021). The Mood State Profile Questionnaire (POMS) determines mood state by negative feelings subscales (Tension, Depression, Anger, Fatigue and Confusion) and positive feeling subscale (Vigor) (McNair et al., 1992). In addition, subscales mood state can be added together (with vigor scored negatively) to determine the total mood disturbance score (TMD) (McNair et al., 1992). On the other hand, total mood disturbance and POMS subscales are reliable predictors of sports performance in several sports (e.g., tennis, football, and triathlon) (Lochbaum et al., 2021). More specifically, the improvement of certain POMS subscales (i.e., tension, depression and fatigue) could promote mental and physical state, which positively affects sports performance in high-level training athletes (Selmi et al., 2023; Stone et al., 2023). Mood state in swimmers could be influenced by training volume. O’Connor et al. (1991) showed that an increase in swimming volume (+47%) negatively affected all mood state subscales. In addition, Pierce (2002) indicated that the mean ratings of both anger and vigor were significantly correlated with training volume (−0.58 and − 0.54, respectively). Based on these ideas, it is necessary to focus on the study of optimal training volume reduction strategies in order to help swimmers be in good psychological and physical condition on the day of the competition.

For this reason, this study aims to investigate the effect of a two-week of tapering period characterized by a gradual reduction in training volume and maintenance of intensity on mood and swimming performance in competitive swimmers. We hypothesized that tapering can improve mood state among swimmers, which could optimize sprint swimming performance.

## Materials and methods

### Study design

**Figure 1:**
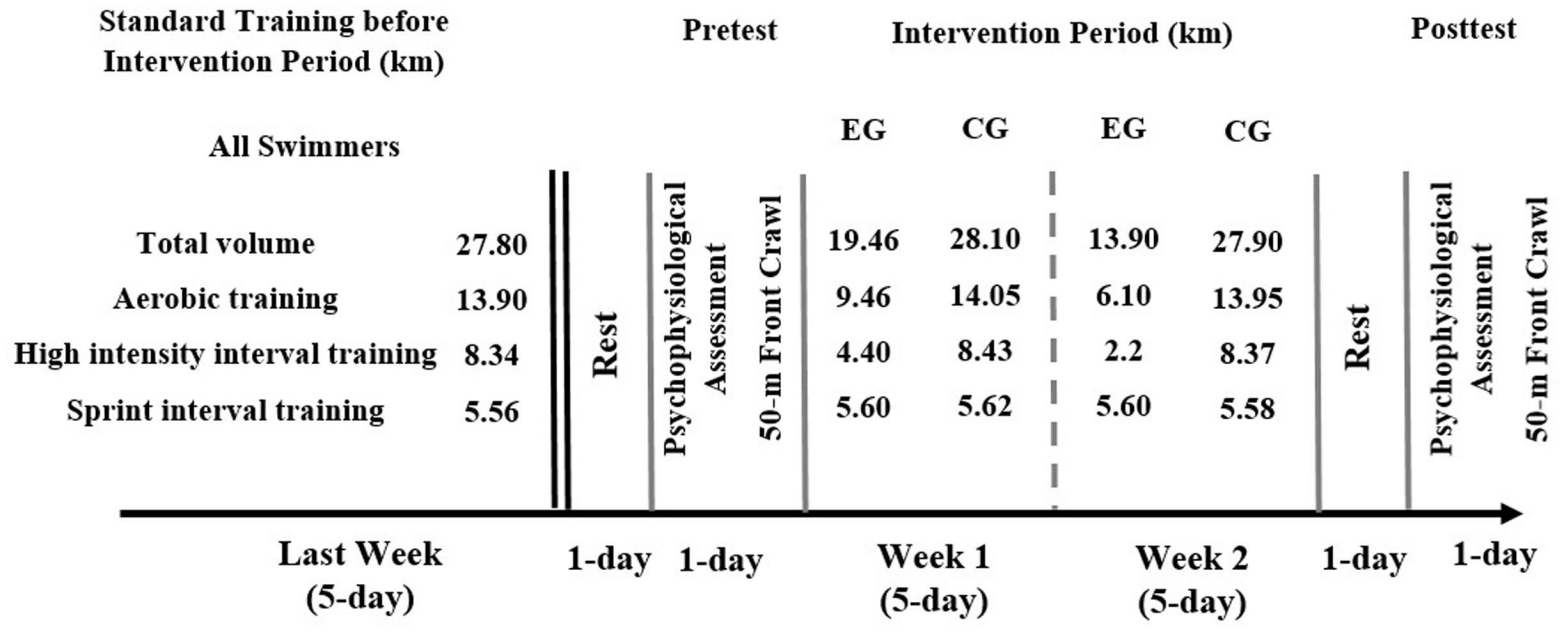
Study design in the both groups. EG: experimental group; CG: control group.

A randomized controlled trial was designed to assess the effect of a two-week tapering period on psychological variables and swimming performance in competitive swimmers, all independent variables were measured before and after the intervention period. All training and swimming tests were performed in a 25-m indoor pool with water and air temperatures of 27.1°C and 25.9°C, respectively, and 64% relative humidity during the sports swimming season (November to December) (Figure 1).

### Participants

Twenty-four male competitive swimmers were randomly assigned into two groups. Experimental group (EG: *n* = 12, age = 16.9 ± 0.52 years; height: 176 ± 8.5 cm; body mass = 73.5 ± 5.80 kg) and control group (*n* = 12, age = 16.1 ± 0.40 years; height: 175 ± 8.6 cm body mass 75.6 ± 5.30 kg). *A priori* power analysis (G*Power 3.1.9.3, Heinrich Heine Universität Düsseldorf, Düsseldorf, Germany) for an independent t test between two groups yielded a sample size of at least 6 swimmers per group to detect large effects (*d* = 1.73), in assuming a power of 0.8 and alpha of 0.05. All swimmers selected were national level and ranked in the top thirty in the 50-crawl test during the last national swim meet and had over 8 years of swimming training. All competitive swimmers had more than 3 years of experience in national level training and competition. The average swimming volume during the last week before the beginning of this present study was 5.56 ± 0.38 km. The best and worst performances achieved in 50 m front crawl were 26.10 and 28.81 s, respectively. Any swimmer who missed more than 10% of training sessions in the last 6 months was excluded from this study. This study was approved by an institutional review board of the Higher Institute of Sport and Physical Education of Ksar Said, University of Manouba, Tunisia (Research Unit of Sports Performance, Health and Society, UR17JS01, protocol code 2022-170), and was carried out according to the latest Helsinki declaration.

### Aquatic training

All aquatic training regardless before or during the intervention period consists of three categories (Chortane et al., 2022; Amara et al., 2023):

1. Aerobic training: low and moderate aerobic training (warm-up, technical drills) i.e., 800 m, 4 × 400 m; at 50 to 75% of maximal heart rate (HRmax).
2. High intensity interval training: Anerobic interval training (front crawl exercise) i.e., 6 × 200 m, 10 × 100 m; at 75 to 85% of HRmax.
3. Sprint interval training: high intensity training (front crawl exercise); i.e., 8 × 25 m, 2 × 6 × (15 m) at >85% of HRmax.

The HR was recorded during the first 10 s after cessation of the bout using a Polar Team 2 (Finland) (Casuso et al., 2017).

### Training before intervention period

All swimmers underwent the same standard training before intervention period (Botonis et al., 2019). Training volume during the last week (5-session) of standard training before intervention was 27.80 km, included approximately 5.56 km of sprint interval training (intensity >85% of HRmax). The mean training load (Mean RPE load) was 683.80 ± 80.24 (Figure 1; Table 1).

**Table 1:** Mean of swimming training volume and training load in the both groups in standard and intervention training periods.

Periods	Weeks	Groups	Mean Distance (m)	Mean Duration (min)	Mean RPE (a.u)	Mean RPE Load (a.u)
Standard training before IP	Last week	All Swimmers	5,560 ± 384.71	92.20 ± 4.77	7.4 ± 0.55	683.80 ± 80.24
Intervention period (IP)	Week 1	EG	3,892 ± 269.30	74.40 ± 3.21	6.00 ± 0.71	448.00 ± 69.86
		CG	5,620 ± 238.75	93.40 ± 4.93	7.20 ± 0.45	673.60 ± 71.35
	Week 2	EG	2,780 ± 192.35	59.80 ± 3.69	5.00 ± 1.00	301.60 ± 76.44
		CG	5,580 ± 216.80	93.00 ± 4.42	6.80 ± 0.84	634.20 ± 99.56

IP, intervention period.

### Intervention period training

**Figure 2:**
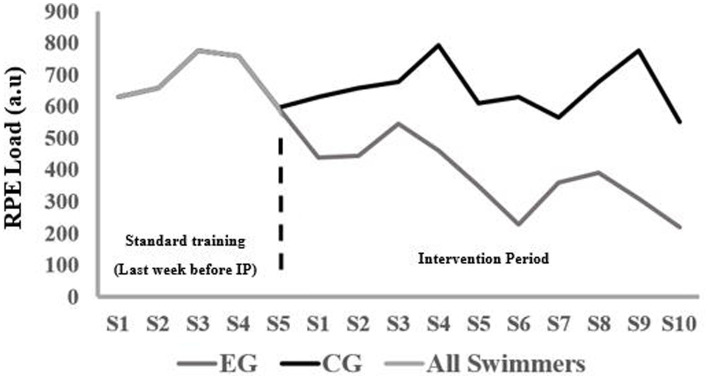
RPE load trajectory in standard training and intervention period in both groups. RPE: Rating of perceived exertion; EG: experimental group; CG: control group; S: session.

The EG was asked to perform aquatic training (5-session per week) characterized by a progressive reduction of 30% total volume during the first week of intervention period (19.46 km) and 50% during the second week (13.90 km) by compared to total volume during the last week of standard training. Maintaining or slightly increasing sprint interval training (5.60 km per week) (Papoti et al., 2007; Botonis et al., 2019). The mean RPE load was decreased during the 2 weeks of intervention period (week 1: 448.00 ± 69.86; week 2: 301.60 ± 76.44). The CG continued their standard training, with no reduction in total volume, sprint interval training, and mean RPE load (Figure 2).

The perceived effort load (RPE load) was calculated during each session (30 min after training) according to the following equation (Borg, 1982; Foster et al., 2001):


RPEload=RPE(1−10scale)×training volume(session duration)

Example: if a swimmer performed a 90 min training session and had an RPE value = 7, then his RPE load is equal to: 90 × 7 = 630.00.

### Mood state measures

To measure the mood state of swimmers before and after the intervention period, a Profile of Mood State (POMS) questionnaire was administered 1 hour before the 50-m front crawl test (McNair et al., 1992). This questionnaire (POMS) is formulated with 65 items and consists of six blocks: tension, depression, anger, vigor, fatigue, and confusion. Response to the questionnaire was rated on a five-point scale: (1) “Not at all,” (2) “A little,” (3) “Moderately,” (4) “Quite a Bit.” and (5) “Extremely.” To calculate the Total Mood Disturbance score simply summing the scores for the five negative mood subscales and subtracting the measure of the Vigor score and adding a constant (100) in order to prevent negative numbers:


$$\begin{array}{c} \mathrm{Total mood disturbance}=\left(\left(\begin{array}{l} \mathrm{Tension}+\mathrm{Depression}\\ +\mathrm{Anger}+\mathrm{Fatigue}\\ +\mathrm{Confusion} \end{array}\right)-\left(\mathrm{Vigor}\right)\right)\\ +100 \end{array}$$

The reliability (ICC) between pre-test and post-test of all the mood state and total mood disturbance variables varied between 0.85 and 0.89.

### Swimming performance test

The 50-m front crawl test was performed in the morning at 10:00 AM. All swimmers performed a standard warm-up consisting of an aerobic training block (600-m) and technical exercises and progressive sprints (10 × 15-m) (Amara et al., 2021). The start of the swim test started with a dive start. Two expert timekeepers measured the time achieved during each 50-m front crawl test using a stopwatch (SEIKO S120-4030, Tokyo, Japan). The intraclass correlation coefficient (ICC) for the pre-test and post-test reliability was 0.89.

### Statistical analysis

All data are presented as mean ± SD and were analyzed using SPSS version 26 for Windows (SPSS Inc., Chicago, IL, United States). Normality and sphericity were tested by Shapiro–Wilk test and Mauchly test, respectively. The reliability of the measurements between test and posttest was measured using the intraclass correlation coefficient (ICC) (Weir, 2005). To calculate the effect of time, group and the time × group interaction a repeated measures ANOVA test was performed. The repeated measures ANOVA was also used to identify the differences between the pretest and the posttest in the two groups (time factor). Bonferroni *post hoc* procedure was applied to locate pairwise differences, only if a significant *F*-value was observed. The difference in volume training between both groups was calculated by the Student’s t-test. Effect size is presented by eta squared (*η*^2^), were 0.01 < η^2^ < 0.06 is defined as a small effect, 0.06 < *η*^2^ < 0.14 is defined as medium and *η*^2^ > 0.14 resemble a large effect.

The relationships between the change in total Mood Disturbance and change in swimming performance (50-m front crawl) for each group were determined by the Pearson product–moment correlation. Therefore, the correlation coefficients were interpreted as: small (0.1 to 0.3), moderate (0.3 to 0.5), large (0.5 to 0.7), very large (0.7 to 0.9), and nearly perfect (0.9 to 1.0) (Hopkins et al., 2009). Statistical significance was set at *p* ≤ 0.05.

## Results

**Figure 3:**
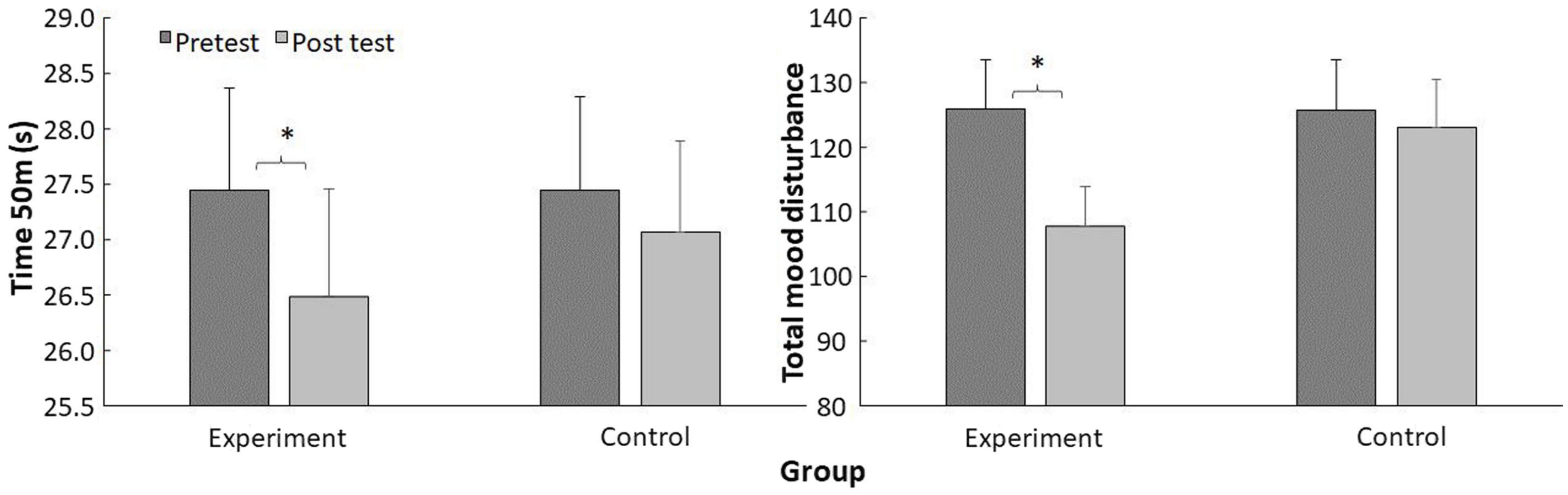
Mean (SD) swimming performance and Total Mood Disturbance before and after two-week of training averaged for the experimental and control group. *Indicate a significant difference from pre- to post test on a *p*<0.05 level.

A significant effect of time (*F* ≥ 1,315, *p* < 0.001, *η*^2^ = 0.99) and interaction effect (*F* ≥ 348, *p* < 0.001, *η*^2^ = 0.94) were found for both swimming performance and total mood disturbance, but only a significant group effect was found for total mood disturbance (*F* = 6.39, *p* = 0.019, *η*^2^ = 0.23) and not for swimming performance (*F* = 0.65, *p* = 0.43, *η*^2^ = 0.03). *Post hoc* comparison revealed a significant improvement in 50 m swimming performance (3.5%) and total mood disturbance (14.4%) the intervention period, while it remained unchanged after standard training (*p* > 0.05) (Table 2; Figure 3).

**Table 2:** Changes in mood state, total mood disturbance and swimming performance after standard and intervention training in the both groups.

Variables	Groups	Pretest	Posttest	Value of *p*	Effect [95% CI]	Delta change (%)
Tension	EG	8.75 ± 2.34	5.92 ± 1.38	0.002	2.83 [1.21 to 4.46]	47.80
	CG	8.92 ± 2.31	8.42 ± 1.96	0.575	0.50 [−1.32 to 2.32]	5.61
Depression	EG	7.25 ± 1.77	4.42 ± 1.17	<0.001	2.83 [1.57 to 4.10]	39.03
	CG	7.33 ± 1.72	7.00 ± 1.86	0.653	0.33 [−1.18 to 1.85]	4.50
Anger	EG	8.42 ± 1.68	6.67 ± 2.19	0.039	1.75 [0.10 to 3.40]	20.78
	CG	8.25 ± 1.82	7.67 ± 1.67	0.421	0.58 [−0.89 to 2.06]	7.03
Fatigue	EG	8.58 ± 2.28	5.08 ± 1.51	<0.001	3.50 [1.87 to 5.13]	40.79
	CG	8.67 ± 2.31	8.25 ± 2.26	0.660	0.42 [−1.52 to 2.35]	4.84
Confusion	EG	6.42 ± 1.51	4.92 ± 1.88	0.042	1.50 [0.06 to 2.94]	23.36
	CG	6.17 ± 1.53	5.83 ± 1.59	0.605	0.33 [−0.99 to 1.65]	5.35
Vigor	EG	13.50 ± 1.78	19.25 ± 1.77	<0.00	−5.75 [−7.25 to −4.25]	42.59
	CG	13.58 ± 1.73	14.17 ± 1.64	0.406	−0.58 [−2.01 to 0.85]	4.27
TMD	EG	125.92 ± 7.63	107.75 ± 6.24	<0.001	18.17 [12.26 to 24.07]	14.43
	CG	125.75 ± 7.84	123.00 ± 7.54	0.391	2.75 [−3.76 to 9.26]	2.19
T 50-m	EG	27.45 ± 0.91	26.49 ± 0.97	0.020	0.96 [0.17 to 1.76]	3.51
	CG	27.45 ± 0.84	27.07 ± 0.81	0.275	0.38 [−0.32 to 1.08]	1.37

TMD, total mood disturbance.

**Figure 4:**
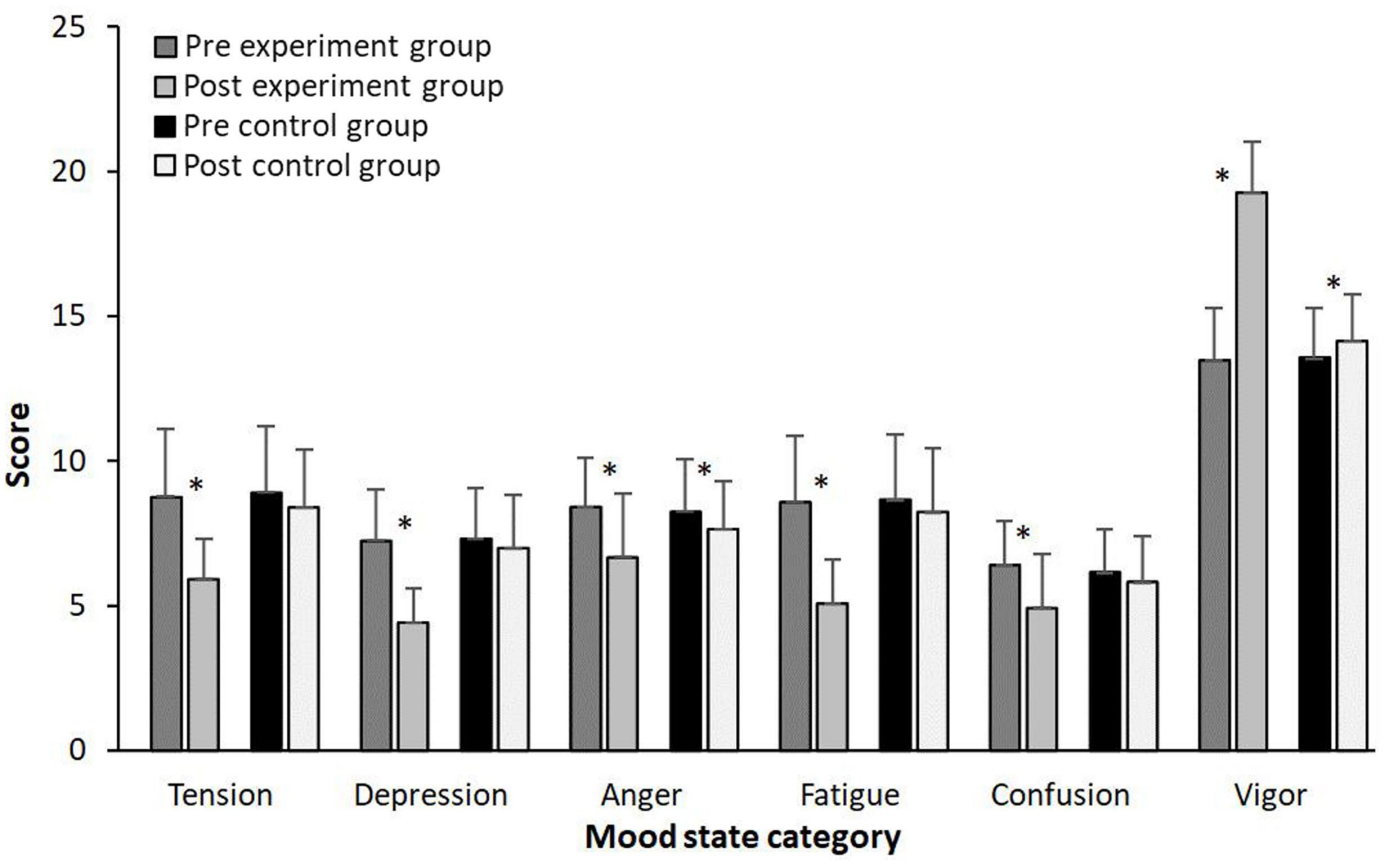
Mean (SD) swimming performance and Total Mood Disturbance before and after two-week of training averaged for the experimental and control group. *Indicate a significant difference from pre- to post test on a *p*<0.05 level.

When analyzing mood state for the different categories a significant effect of time in the all of mood subscales (*F* ≥ 38, *p* < 0.001, *η*^2^ ≥ 0.63) and interaction effect (*F* ≥ 15.4, *p* < 0.001, *η*^2^ = 0.41) were found, while only a significant group effect was found for the subscale vigor (*F* = 12.7, *p* = 0.002, *η*^2^ = 0.37). *Post hoc* comparison revealed that in the experimental group all negative mood subscales (tension, depression, anger, fatigue and confusion) decreased (≥20%) after 2 weeks of tapering period, while significant increase in the state of vigor was observed (42.6%), while in the control group only small significant increases in vigor (4.2%) and decrease in anger (7.0%) were observed (Figure 4).

### Training load and rating of perceived exertion

**Figure 5:**
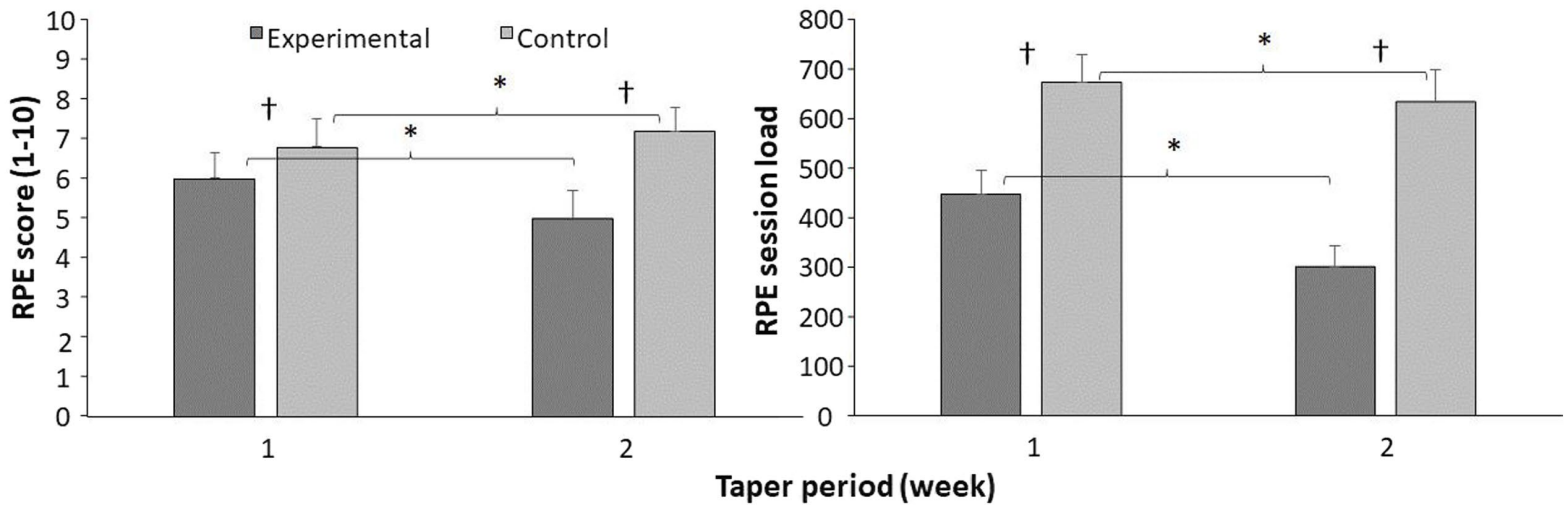
Mean (SD) rating of perceived exertion (RPE) and RPE session load per week during tapering period for each group. *Indicates a significant change for this group from week 1 to 2. ^†^Indicates a significant between the groups on a *p*<005 level.

A significant difference was observed in RPE score and RPE session over the 2 weeks of tapering, together with a between group and interaction effect (*F* ≥ 11.4, *p* ≤ 0.003, *η*^2^ ≥ 0.34). *Post hoc* comparison revealed that RPE score significantly decreased for the experimental group, while it increased for the control group from week 1 to 2 decreased and that the RPE session load decreased for both groups in the same period. Furthermore, were the RPE and RPE session load significantly higher for the control group compared with the experimental group each week (Figure 5).

### Relationship between change swimming performance and the total mood disturbance

**Figure 6:**
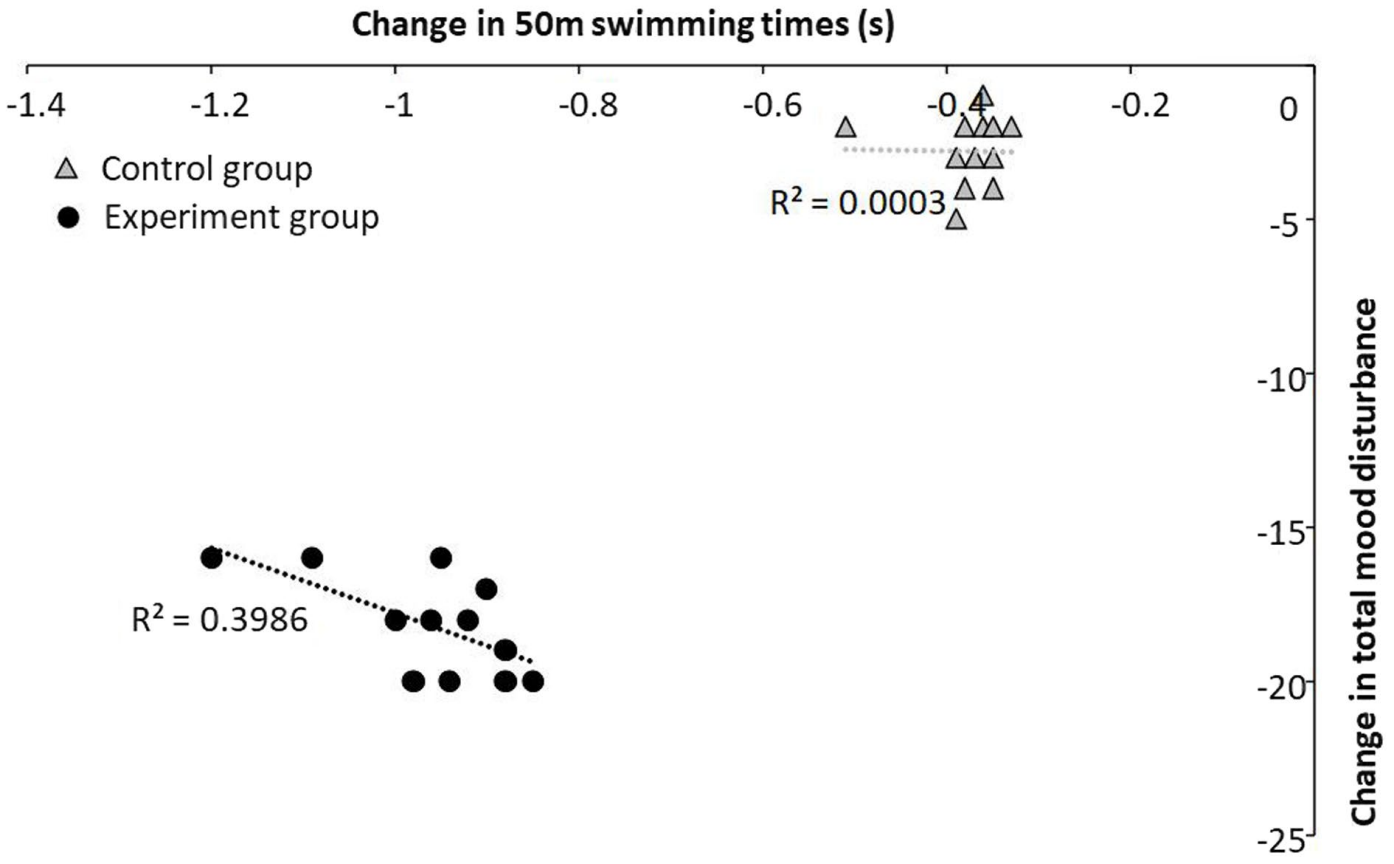
Relationship between change in swimming performance and change in total mood disturbance for each group.

The results of the present study revealed that the change in total mood disturbance had a large significant correlation with the change in swimming performance (*r* = −0.63, *p* = 0.028) for the experiment group, while for the control group no significant correlation between the two variables was found (*r* = −0.017, *p* = 0.95, Figure 6).

## Discussion

The purpose of this study was to investigate the effect of a tapering period on psychological state and swimming performance in competitive swimmers. Our findings revealed that 2 weeks of tapering decreased RPE and RPE session load, improved the different mood state categories, total mood disturbance and swimming performance in competitive swimmers. Furthermore, a significant correlation between change in swimming performance and total mood distribution was found for the tapering group.

The progressive reduction in the training load (RPE load) (34.5 to 55.9%) and in the volume training (30 to 50%) during the 2 weeks of tapering had reduced the rating score of RPE (18.9 to 33.4%) (Table 1), which positively influenced mood state (20.8 to 47.8%) and total mood disturbance (14.4%) in competitive swimmers. These results are in line with Jafer et al. (2019), who revealed that two models of tapering strategies (i.e., high intensity-low volume and high intensity-moderate volume) have important effects in the improvement of the total mood disturbance (23.87 to 36.82%). Jafer et al. (2019) indicated that the increase in Vigor-Activity and the decrease in Aggression-Hostility and Confusion-Bewilderments decreased the total mood disturbance score, which causes an improvement in mood state in runners athletes aged 20 ± 2.0 years. In the same context, Filaire et al. (2001) showed that mood state increase proportionally with increasing training load. The onset of fatigue during periods of high-volume training led to decreased Vigor-activity, which negatively affects the moods of competitive athletes (Filaire et al., 2001; Bachelor et al., 2023). The total mood disturbance and all mood state variables remains unchanged after habitual training, supporting the idea that the tapering period characterized by a decrease in training volume and training load could positively affect the psychological state of competitive swimmers.

Our results revealed significant correlation (*r* = −0.63) between total mood disturbance and swimming performance in the experiment group. In fact, improving mood (i.e., tension, depression and fatigue) could positively affect cognitive (e.g., concentration, attention, reflex) and somatic (physical freshness, energy, resistance to fatigue) abilities, which promotes fitness in competitive athletes (Coutts et al., 2007a,b; Stone et al., 2023). In addition, the increase in vigor and total mood disturbance could be motivating factors during the physical performance test (Sahli et al., 2020; Aydi et al., 2022). In fact, a positive mood state can lead to the avoidance of mental blocks during physical training, which promotes performance and reduces the risk of muscle injury (i.e., disturbance in muscle contractions) (Stults-Kolehmainen and Sinha, 2014; Basso and Suzuki, 2017).

Our findings showed that swimming performance was improved after 2 weeks of tapering (3.5%). Our results are consistent with the results of Houmard et al. (1994) who revealed that a tapering period (7 to 21 days) characterized by a gradual reduction in training volume (60 to 90%) and daily high intensity training improved swimming performance (3%). Houmard et al. (1994) showed that the positive consequences of a tapering period (i.e., increase in muscle power, restoration of plasma hematocrit and hemoglobin) could express this improvement in swimming performance. In fact, Tapering could cause positive effects on aerobic capacity, improved ventilatory threshold and a gain in muscle glycogen concentration (35%), which positively affects physical performance (Neary et al., 1992). Swimming performance was unaltered after usual training, which confirms the idea that a period of progressive reduction could improve the physical and mental state, which causes an improvement in swimming performance in competitive swimmers (Stone et al., 2023).

In summary, this present study showed that 2 weeks of tapering positively affected various mood subscales, resulting in improved psychological states and TMD in competitive swimmers. In addition, a significant correlation was observed between the total mood disturbance and the swimming performance in experiment group, which confirms that the variation in the TMD could affect the swimming performance (Houmard et al., 1994; Botonis et al., 2019). Moreover, a progressive reduction in training volume with a maintain of intensity could decrease psychological and physical fatigue, which helps the swimmer to overcome the negative influences of overtraining (i.e., mental blockage, injury and muscle fatigue) which causes an improvement in swimming performance (Botonis et al., 2019; Stone et al., 2023).

Certain limitations appear in this study. For instance, the presence of other information on physiological adaptations such as muscular adaptation (i.e., strength gain, muscle oxygenation) and cardiorespiratory adaptation (e.g., ventilatory threshold, heart rate) during and after the tapering period could shed further light on the effects of tapering in competitive swimmers. Additionally, future research should investigate the effect of tapering influences physical and mental status at the level of other age categories (i.e., prepubescents, master swimmers).

## Conclusion

Our findings revealed that a two-week of tapering period characterized by a progressive reduction in volume training and maintain of intensity affected positively various mood subscales, which improved psychological state in competitive swimmers. A significant correlation between TMD and swimming performance after tapering explained that a change in the mood state affect the swimming performance. Swimming coaches are advised to included tapering according to the standards we mentioned earlier before competitive swimming to decrease mental and physical fatigue, which helps the swimmers overcome the negative influences of overtraining and therefore they can promote sprint-swimming performance.

## Data availability statement

The original contributions presented in the study are included in the article/supplementary material, further inquiries can be directed to the corresponding author.

## Ethics statement

The studies involving humans were approved by Institutional review board of the Higher Institute of Sport and Physical Education of Ksar Said, University of Manouba, Tunisia. The studies were conducted in accordance with the local legislation and institutional requirements. Written informed consent for participation in this study was provided by the participants' legal guardians/next of kin.

## Author contributions

HA: Conceptualization, Methodology, Writing – original draft. SA: Conceptualization, Methodology, Writing – original draft. HR: Formal analysis, Writing – original draft, Writing – review & editing. TB: Formal analysis, Writing – original draft. RT: Supervision, Writing – original draft, Writing – review & editing.
